# The association between preserved ratio impaired spirometry and adverse outcomes of depression and anxiety: evidence from the UK Biobank

**DOI:** 10.1017/S0033291724002162

**Published:** 2024-09

**Authors:** Kai Yang, Lingwei Wang, Jun Shen, Shuyu Chen, Yuanyuan Liu, Rongchang Chen

**Affiliations:** 1 Department of Pulmonary and Critical Care Medicine, Shenzhen Institute of Respiratory Diseases, Shenzhen People's Hospital (First Affiliated Hospital of Southern University of Science and Technology, Second Clinical Medical College of Jinan University), Shenzhen, 518001, China; 2 Department of Orthopedics, the Seventh Affiliated Hospital, Sun Yat-Sen University, Shenzhen, 518000, China

**Keywords:** anxiety, depression, epidemiology, lung function, preserved ratio impaired spirometry

## Abstract

**Background:**

Preserved ratio impaired spirometry (PRISm) is a new lung function impairment phenotype and has been recognized as a risk factor for various adverse outcomes. We aimed to examine the associations of this new lung function impairment phenotype with depression and anxiety in longitudinal studies.

**Methods:**

We included 369 597 participants from the UK Biobank cohort, and divided them into population 1 without depression or anxiety and population 2 with depression or anxiety at baseline. Cox proportional hazard models were performed to evaluate the associations of lung function impairment phenotype with adverse outcomes of depression and anxiety, as well as their subtypes.

**Results:**

At baseline, 38 879 (10.5%) participants were diagnosed with PRISm. In population 1, the adjusted hazard ratios (HRs) for PRISm (*v.* normal spirometry) were 1.12 (95% CI 1.07–1.18) for incident depression, and 1.11 (95% CI 1.06–1.15) for incident anxiety, respectively. In population 2, PRISm was a risk factor for mortality in participants with depression (HR: 1.46; 95% CI 1.31–1.62) and anxiety (HR: 1.70; 95% CI 1.44–2.02), compared with normal spirometry. The magnitudes of these associations were similar in the phenotypes of lung function impairment and the subtypes of mental disorders. Trajectory analysis showed that the transition from normal spirometry to PRISm was associated with a higher risk of mortality in participants with depression and anxiety.

**Conclusions:**

PRISm and airflow obstruction have similar risks of depression and anxiety. PRISm recognition may contribute to the prevention of depression and anxiety.

## Introduction

Depression and anxiety are common mental disorders associated with increased comorbidities, premature death, and healthcare costs (Charlson et al., 2019; Patel et al., 2018). According to the Global Burden of Disease 2019 and World Health Organization report, the prevalences of depression and anxiety are 3.4 and 3.8%, which account for 7.5 and 3.4% of global disability, respectively (GBD, 2019 Mental Disorders Collaborators, 2022; World Health Organization, 2017). Depression and anxiety have been reported to co-occur with chronic obstructive pulmonary disease (COPD), which is the most common chronic lung disease with progressive lung function decline (Cavaillès et al., 2013; Yohannes & Alexopoulos, 2014). Previous studies have also indicated that COPD was an independent risk factor for depression and anxiety, and the prevalences of depression and anxiety in COPD patients were reported to be as high as 50% (Matte et al., 2016; Pumar et al., 2014; Riblet, Gottlieb, Hoyt, Watts, & Shiner, 2020). Given the severity of depression and anxiety in obstructive lung function decline, the potential causal associations of non-obstructive lung function abnormalities with depression and anxiety also merit exploration in prospective studies.

In population-based studies, 7.1 to 20.3% of individuals undergoing spirometry had proportional declines in forced expired volume in the first second (FEV_1_) and forced vital capacity (FVC), normal FEV_1_ to FVC ratio, and decreased FEV_1_% predicted (Wan, 2022). This pattern of lung function impairment is defined as preserved ratio impaired spirometry (PRISm), which is often neglected in previous studies (Wan et al., 2014). Recently, PRISm is increasingly recognized to have a clinically important role in the health of the general population (Godfrey & Jankowich, 2016). Several population-based studies have shown that PRISm was related to increased respiratory symptoms, higher incidence of comorbidities and greater mortality, compared to normal spirometry (Higbee, Granell, Davey Smith, & Dodd, 2022; Li et al., 2023b; Wan et al., 2021; Washio et al., 2022; Zheng et al., 2023).

The relationships of COPD with depression and anxiety have been reported previously (Yohannes & Alexopoulos, 2014). Although not fully elucidated, some factors have been reported to contribute to the development of depression and anxiety in COPD patients. COPD patients have higher smoking exposure, more respiratory symptoms, reduced exercise capacity and more comorbidities, contributing to depression and anxiety (Huang et al., 2021). Chronic systemic inflammation may also lead to psychological distress in patients with COPD (Lu et al., 2013). However, the risks of depression and anxiety in individuals with PRISm remain to be elucidated.

To address these knowledge gaps, we aimed to use data from the UK Biobank to investigate the associations of baseline PRISm with adverse outcomes of depression and anxiety during follow-up, as well as their subtypes. We also assessed the associations of lung function trajectories with depression and anxiety in a subpopulation with repeated spirometry.

## Methods

### Study population

The UK Biobank study is a nationwide prospective study of approximately 500 000 middle-aged and older adults in the UK (Sudlow et al., 2015). The participants were recruited between 19 Dec 2006 and 10 Oct 2010, and baseline data on sociodemographic characteristics, lifestyle, physical measurements and health-related outcomes were collected through brief interviews, questionnaires and linkage to national health records. Participants with missing information on spirometry, depression, anxiety, or covariates were excluded from the study.

Three main populations were identified in this study (online Supplementary Fig. S1). In population 1, we included participants without depression or anxiety at baseline. This population was used to explore the risks of baseline lung function categories on incident depression or anxiety during follow-up. In population 2, participants with depression or anxiety at baseline were included to investigate the association of baseline lung function categories with mortality during follow-up. The relationships of lung function trajectories with depression and anxiety were evaluated in the subpopulation with repeated spirometry.

The research protocol for this study has been approved by the review committee of UK Biobank. The study was approved by the North-West Research Ethics Committee and all participants provided written informed consent before enrollment.

### Assessments of spirometry

The participants were requested to complete 2–3 pre-bronchodilator spirometry at recruitment if they consented and had no contraindications. Participants were invited for repeated spirometry between 30 Apr 2014, and 13 Mar 2020 if they resided close to an assessment center. The highest FEV_1_ and FVC values from acceptable blows were used in this study.

PRISm was defined as FEV_1_/FVC of 0.70 or higher and FEV_1_ of less than 80% predicted. Airflow obstruction (AO) was defined as FEV_1_/FVC of less than 0.70 according to the Global Initiative for Chronic Obstructive Lung Disease (GOLD) (Agustí et al., 2023). A FEV_1_/FVC of 0.70 or higher and a FEV_1_ of 80% predicted or higher were considered normal spirometry. Predicted FEV_1_ was calculated according to the Global Lung Initiative (GLI) established prediction equations considering race, gender, age and height (Quanjer et al., 2012). FEV_1_% value was used to evaluate the severity of lung function impairment. We categorized lung function at baseline into three groups: normal spirometry, PRISm and AO, and lung function trajectories into nine groups: normal to normal, normal to PRISm, normal to AO, PRISm to PRISm, PRISm to AO, PRISm to normal, AO to AO, AO to PRISm, and AO to normal.

### Outcome measurement and covariates

The outcome of population 1 was time to incident depression, anxiety or the subtypes, and the outcome of population 2 was the time to mortality. Depression and anxiety were diagnosed by medical doctors according to the guidelines of the National Institute for Health and Care Excellence. The assessments were conducted using records derived from death register, primary care, hospital admission, and self-report information. Depression was defined by the first record of five subtypes: depressive episode (F32), recurrent depressive disorder (F33), persistent mood disorder (F34), other mood disorder (F38) or unspecified mood disorder (F39), coded by International Classification of Diseases version-10 (ICD-10). Anxiety included phobic anxiety disorders (F40) and other anxiety disorders (F41).

Based on previous research on risk factors for depression and anxiety in UK Biobank participants, several potential confounders were considered as covariates in this study (Gao et al., 2023; Yang, Wang, Huang, Kelly, & Li, 2023). Age, sex (female and male), ethnicity (White, Black, and other), smoking status (never, previous, and current), drinking status (never, previous, and current), and income level (<£ 31 000 and ⩾£ 31 000) were collected using baseline questionnaires. Height and weight were obtained through physical examinations, and body mass index (BMI) was calculated as weight (kg)/height (m)^2^. Physical activity was evaluated by the number of days/week that the participant walked for 10+ minutes, and categorized into 0 days/week, 1–3 days/week and 3–7 days/week. Hypertension was defined by ICD-10 codes I10–I13 and I15, diabetes by E10–E14, and cardiovascular disease (CVD) by I5–I9, I11, I13, I20–I28 and I30–I52. The variable assignments for these covariates were provided in online Supplementary Table S1.

### Statistical analysis

Baseline characteristics were presented as the number of observations and percentage for categorical variables and mean and standard deviation (s.d.) for continuous variables. The demographic differences between participants with PRISm and those with normal spirometry or AO were compared using Pearson's χ^2^ test or *t* test as appropriate.

Cumulative survival curves were calculated using the Kaplan-Meier method grouped by lung function categories and compared using the log-rank test. The Cox proportional hazard model was used to estimate the hazard ratio (HR) with a 95% confidence interval (CI) for incident depression, anxiety or subtypes in population 1 and mortality in population 2, in relationship to lung function categories at baseline and lung function trajectories. The non-linear associations between FEV_1_% predicted and outcomes were also investigated with penalized cubic splines fitted in Cox proportional hazard models (online Supplementary Method) (Govindarajulu, Malloy, Ganguli, Spiegelman, & Eisen, 2009). The coefficient curves of FEV_1_% predicted were zeroed at 80% (HR = 1) in the non-linear models. Likelihood ratio tests were used to test the statistical significance of overall and non-linear part of FEV_1_% predicted.

Statistical analyses were performed using SAS 9.4 (SAS Institute Inc, Cary, North Carolina) and R 4.2.1 (R Foundation for Statistical Computing, Vienna). Two-sided values of *p* < 0.05 were considered statistically significant.

## Results

### Baseline characteristics

Of the participants in the UK Biobank, 369 597 were included in this study, in which the prevalences of PRISm and AO at baseline were 10.5 and 14.7%, respectively. The participant characteristics by baseline lung function categories were presented in Table 1. Participants with PRISm were older, were less likely to be White, had higher BMI, were more likely to be current smokers, were less likely to be current drinkers, had less physical activity, had lower income level, and had higher prevalences of hypertension, diabetes and CVD than those with normal spirometry.

**Table 1.** Demographic information of the total population in this study

Characteristic	Normal	PRISm	AO
***N***	276 378	38 879	54 340
**Age** ^a^^b^	55.4 ± 8.1	55.7 ± 8.1	58.5 ± 7.7
Sex ^b^			
Male	126 410(45.7)	17 901(46.0)	30 851(56.8)
Female	149 968(54.3)	20 978(54.0)	23 489(43.2)
Ethnicity ^a^^b^			
White	265 763(96.2)	34 873(89.7)	52 218(96.1)
Black	3351(1.2)	1046(2.7)	646(1.2)
Other	7264(2.6)	2960(7.6)	1476(2.7)
**BMI** ^a^^b^	27.2 ± 4.5	29.0 ± 5.6	26.6 ± 4.5
Smoking status ^a^^b^			
Never	159 559(57.7)	20 948(53.9)	23 583(43.4)
Previous	94 340(34.1)	13 177(33.9)	20 813(38.3)
Current	22 479(8.1)	4754(12.2)	9944(18.3)
Drinking Status ^a^^b^			
Never	9051(3.3)	2371(6.1)	1923(3.5)
Previous	8122(2.9)	1745(4.5)	2065(3.8)
Current	259 205(93.8)	34 763(89.4)	50 352(92.7)
Physical activity ^a^^b^			
0 days/week	33 799(12.2)	6256(16.1)	6881(12.7)
1–2 days/week	108 383(39.2)	14 989(38.6)	19 605(36.1)
3–7 days/week	134 196(48.6)	17 634(45.4)	27 854(51.3)
Income level ^a^^b^			
< 31 000	120 541(43.6)	20 415(52.5)	30 470(56.1)
31 000~	155 837(56.4)	18 464(47.5)	23 870(43.9)
**FEV_1_/FVC** ^a^^b^	78.3 ± 4.3	76.8 ± 4.8	64.0 ± 6.7
**FEV_1_ % predicted** ^a^^b^	99.7 ± 12.4	71.9 ± 8.2	80.3 ± 20.8
**Hypertension at baseline** ^a^^b^	64 381(23.3)	12 774(32.9)	15 270(28.1)
**Diabetes at baseline** ^a^^b^	5632(2.0)	2079(5.4)	1500(2.8)
**CVD at baseline** ^a^^b^	18 770(6.8)	4726(12.2)	5691(10.5)

a *p* < 0.05 for the comparison of PRISm and normal spirometry.

b *p* < 0.05 for the comparison of PRISm and AO.

*Note:* Age, BMI and lung function were presented as mean ± standard deviation, and other characteristics as number (percentage).

The prevalences of depression (9.3%), depressive episode (8.9%), recurrent depressive disorder (0.6%), persistent mood disorder (0.2%), anxiety (3.9%) and other anxiety disorder (3.7%) were significantly higher in participants with PRISm than in those with normal spirometry and AO (online Supplementary Table S2). In population 1, the incidences of depression, depressive episode, anxiety, phobic anxiety disorder and other anxiety disorder in participants with PRISm were 5.3 5.2, 5.5, 0.7 and 5.1% after a median follow-up period of 13.9 years, which were also significantly higher than those in the other two groups. In population 2, the mortality rates of participants with depression (12.5%), depressive episode (12.3%), recurrent depressive disorder (13.5%), anxiety (12.2%) and other anxiety disorder (12.0%) were significantly higher than those with normal spirometry, and lower than those with AO.

### Incident depression and anxiety by lung function categories

**Figure 1.**
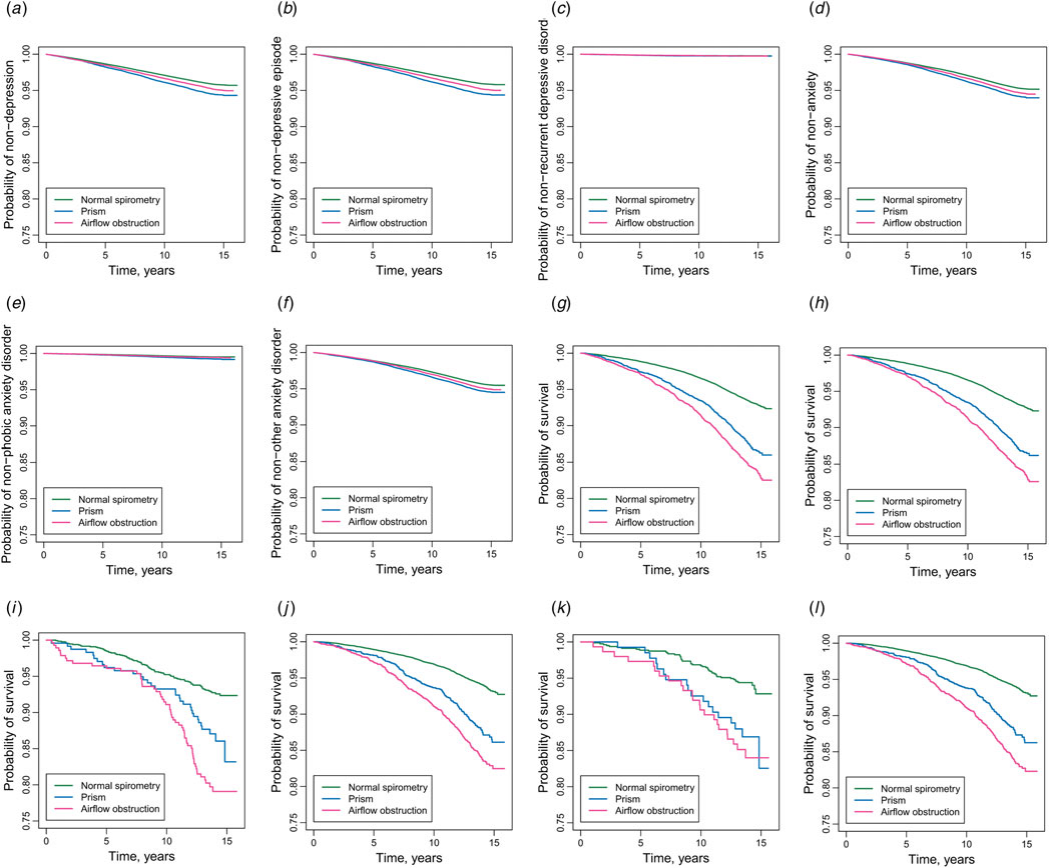
Kaplan-Meier curves of incident depression, anxiety and mortality by baseline lung function categories in different populations. (a) Depression in population 1. (b) Depressive episode in population 1. (c) Recurrent depressive disorder in population 1. (e) Anxiety in population 1. (e) Phobic anxiety disorder in population 1. (f) Other anxiety disorder in population 1. (g) Mortality of participants with depression in population 2. (h) Mortality of participants with depressive episode in population 2. (i) Mortality of participants with recurrent depressive disorder in population 2. (j) Mortality of participants with anxiety in population 2. (k) Mortality of participants with phobic anxiety disorder in population 2. (l) Mortality of participants with other anxiety disorder in population 2.

As presented in Fig. 1, participants with PRISm had the highest unadjusted incidences of depression (Figs 1a to c) and anxiety (Figs 1d to 1f). Table 2 showed the associations of baseline lung function categories with incident depression and anxiety in Cox proportional hazard models. In the multivariate-adjusted models, the risk of depression was 12% higher in participants with PRISm compared to those with normal spirometry (HR: 1.12; 95% CI 1.07–1.18), and the increased risk was 11% for anxiety (HR: 1.11; 95% CI 1.06–1.15). The higher risks of PRISm were significant in the subtypes of depression episode (HR: 1.14; 95% CI 1.08–1.20), phobic anxiety disorder (HR: 1.35; 95% CI 1.18–1.54) and other anxiety disorder (HR: 1.10; 95% CI 1.04–1.15).

**Table 2.** Hazard ratios (95% CIs) of incident depression and anxiety with different baseline lung function categories in individuals without depression and anxiety

Event	Category	Crude model		Adjusted model^a^	
		HR (95% CI)	*p*	HR (95% CI)	*p*
Depression	Normal	Reference		Reference	
	PRISm	1.34(1.28–1.41)	<0.01	1.12(1.07–1.18)	<0.01
	AO	1.16(1.11–1.22)	<0.01	1.12(1.07–1.17)	<0.01
Depressive episode	Normal	Reference		Reference	
	PRISm	1.36(1.30–1.43)	<0.01	1.14(1.08–1.20)	<0.01
	AO	1.18(1.13–1.23)	<0.01	1.13(1.08–1.18)	<0.01
Recurrent depressive disorder	Normal	Reference		Reference	
	PRISm	1.19(0.96–1.48)	0.11	1.04(0.84–1.30)	0.72
	AO	1.14(0.94–1.37)	0.19	1.07(0.88–1.30)	0.50
Persistent mood disorder	Normal	Reference		Reference	
	PRISm	1.36(0.89–2.08)	0.16	1.13(0.73–1.75)	0.59
	AO	1.09(0.72–1.63)	0.70	1.02(0.67–1.56)	0.92
Other mood disorder	Normal	Reference		Reference	
	PRISm	0.95(0.41–2.22)	0.90	0.72(0.30–1.71)	0.45
	AO	1.69(0.94–3.03)	0.08	1.60(0.88–2.93)	0.13
Unspecified mood disorder	Normal	Reference		Reference	
	PRISm	1.29(0.92–1.81)	0.14	1.14(0.81–1.61)	0.46
	AO	0.71(0.49–1.04)	0.08	0.77(0.53–1.13)	0.18
Anxiety	Normal	Reference		Reference	
	PRISm	1.26(1.20–1.32)	<0.01	1.11(1.06–1.17)	<0.01
	AO	1.14(1.09–1.18)	<0.01	1.10(1.06–1.15)	<0.01
Phobic anxiety disorder	Normal	Reference		Reference	
	PRISm	1.61(1.41–1.83)	<0.01	1.35(1.18–1.54)	<0.01
	AO	1.16(1.02–1.32)	0.03	1.10(0.97–1.26)	0.15
Other anxiety disorder	Normal	Reference		Reference	
	PRISm	1.23(1.17–1.29)	<0.01	1.10(1.04–1.15)	<0.01
	AO	1.13(1.08–1.18)	<0.01	1.10(1.05–1.15)	<0.01

a Adjusted for age, sex, ethnicity, BMI, smoking status, drinking status, income level, physical activity, hypertension, diabetes and CVD.

**Figure 2.**
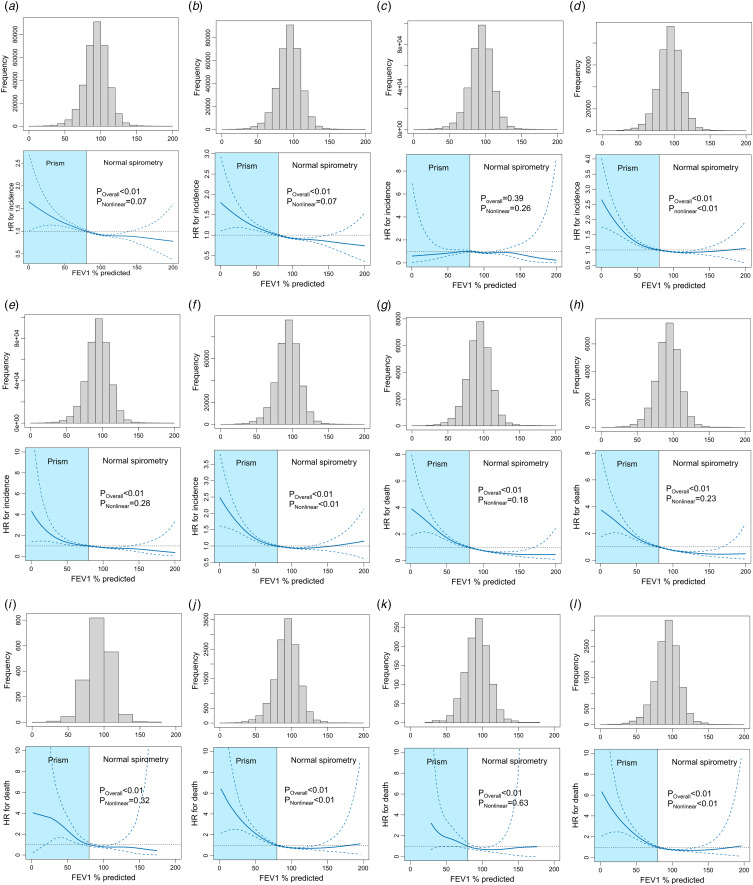
Distribution of FEV_1_% predicted and penalized cubic spline analyses for the association of FEV_1_% predicted with incident depression and anxiety. (a) Depression in population 1. (b) Depressive episode in population 1. (c) Recurrent depressive disorder in population 1. (d) Anxiety in population 1. (e) Phobic anxiety disorder in population 1. (f) Other anxiety disorder in population 1. (g) Mortality of participants with depression in population 2. (h) Mortality of participants with depressive episode in population 2. (i) Mortality of participants with recurrent depressive disorder in population 2. (j) Mortality of participants with anxiety in population 2. (k) Mortality of participants with phobic anxiety disorder in population 2. (l) Mortality of participants with other anxiety disorder in population 2.

The non-linear associations of FEV_1_% predicted with incident depression and anxiety in participants without AO were shown in Fig. 2. The FEV_1_% predicted of more than 99% of these participants ranged between 54% and 140%. The non-linear association between FEV_1_% predicted and incident depression was marginally significant (*p*_Nonlinear_ = 0.07), which was mainly contributed by depressive episode (Figs 2a to c). Furthermore, we found a non-linear increase of anxiety risk when FEV_1_% predicted decreased in participants with PRISm (*p*_Nonlinear_ < 0.01), which was mainly contributed by other anxiety disorder (Figs 2d to f).

### Mortality of participants with depression and anxiety by lung function categories

The associations of baseline lung function categories with the mortality of participants with depression and anxiety were presented in Fig. 1 and Table 3. The unadjusted mortality rates for PRISm were intermediate between normal spirometry and AO among participants with depression (Figs 1g to i) and anxiety (Figs 1j to l). In Cox proportional hazard models adjusted for potential confounders, PRISm increased 46 and 70% of mortality rates in participants with depression (HR: 1.46; 95% CI 1.31–1.62) and anxiety (HR: 1.70; 95% CI 1.44–2.02) (Table 3). The higher risks of mortality in participants with PRISm were observed in the subtypes of depression episode (HR: 1.43; 95% CI 1.28–1.60) and other anxiety disorder (HR: 1.68; 95% CI 1.41–2.00). The non-linear association between FEV_1_% predicted and mortality was not statistically significant in participants with depression (*p*_Nonlinear_ = 0.18) (Figs 2g to i), but it was statistically significant in those with anxiety and other anxiety disorder (*p*_Nonlinear_ < 0.01) (Figs 2j to 2l).

**Table 3.** Hazard ratios (95% CIs) of all-cause mortality with different baseline lung function categories in individuals with depression and anxiety

Event	Category	Crude model		Adjusted model^a^	
		HR (95% CI)	*p*	HR (95% CI)	*p*
Depression	Normal	Reference		Reference	
	PRISm	1.95(1.75–2.16)	<0.01	1.46(1.31–1.62)	<0.01
	AO	2.45(2.23–2.68)	<0.01	1.56(1.42–1.71)	<0.01
Depressive episode	Normal	Reference		Reference	
	PRISm	1.92(1.72–2.14)	<0.01	1.43(1.28–1.60)	<0.01
	AO	2.42(2.21–2.66)	<0.01	1.55(1.41–1.71)	<0.01
Recurrent depressive disorder	Normal	Reference		Reference	
	PRISm	1.93(1.29–2.88)	<0.01	1.27(0.83–1.93)	0.27
	AO	2.98(2.15–4.14)	<0.01	1.88(1.32–2.68)	<0.01
Persistent mood disorder	Normal	Reference		Reference	
	PRISm	1.28(0.56–2.95)	0.56	1.03(0.42–2.54)	0.95
	AO	2.16(1.10–4.27)	0.03	1.38(0.64–2.96)	0.41
Other mood disorder	Normal	Reference		Reference	
	PRISm	6.81(1.23–37.57)	0.03	0.79(0.02–40.51)	0.90
	AO	1.69(0.19–15.17)	0.64	0.46(0.01–33.67)	0.72
Unspecified mood disorder	Normal	Reference		Reference	
	PRISm	2.17(0.86–5.50)	0.1	1.78(0.68–4.69)	0.24
	AO	2.46(0.97–6.25)	0.06	1.16(0.37–3.61)	0.80
Anxiety	Normal	Reference		Reference	
	PRISm	2.11(1.79–2.49)	<0.01	1.70(1.44–2.02)	<0.01
	AO	2.80(2.44–3.22)	<0.01	1.79(1.55–2.07)	<0.01
Phobic anxiety disorder	Normal	Reference		Reference	
	PRISm	2.33(1.36–4.02)	<0.01	1.71(0.97–3.03)	0.07
	AO	2.72(1.65–4.47)	<0.01	1.62(0.95–2.74)	0.08
Other anxiety disorder	Normal	Reference		Reference	
	PRISm	2.07(1.74–2.46)	<0.01	1.68(1.41–2.00)	<0.01
	AO	2.82(2.45–3.25)	<0.01	1.81(1.56–2.10)	<0.01

a Adjusted for age, sex, ethnicity, BMI, smoking status, drinking status, income level, physical activity, hypertension, diabetes and CVD.

### Characteristics of different lung function trajectories

Of the participants included in this study, 35 319 were invited to undergo repeated spirometry after an average of 9.5 years. Among 2654 participants with PRISm at baseline, 1172 (44.2%) reverted to normal spirometry, 1137 (42.8%) had persistent PRISm, and 345 patients (13.0%) developed AO (online Supplementary Fig. S2). Participants with PRISm had a higher risk of developing AO than those with normal spirometry (13.0% *v.* 9.4%; *p* < 0.01).

Participants who transitioned from normal spirometry to PRISm were more likely to be women, had higher BMI, were more likely to be current smokers, were less likely to be White, were less likely to be current drinkers, had less physical activity, had lower income level, and had higher prevalences of hypertension, diabetes and CVD than participants with persistent normal spirometry (*p* < 0.05) (online Supplementary Table S3). Participants with persistent PRISm were less likely to be White and current drinkers, had lower income level, and had higher prevalences of hypertension and diabetes than those who reverted to normal spirometry (*p* < 0.05). Compared with participants who transitioned from PRISm to AO, those with persistent PRISm were less likely to be White, and had higher BMI (*p* < 0.05). Among the participants with normal spirometry at baseline, those with lower FEV_1_/FVC and FEV_1_% predicted tended to transition to PRISm (*p* < 0.05). The participants with PRISm at baseline who had a higher level of FEV_1_% predicted were more likely to transition to normal spirometry, and those who had a lower level of FEV_1_/FVC tended to develop AO (*p* < 0.05).

The prevalences of depression and anxiety at baseline and mortality rates of participants with depression and anxiety were also statistically different among nine lung function trajectories (*p* < 0.05) (online Supplementary Table S4).

### Depression and anxiety by lung function trajectories

After a median follow-up period of 4.4 years, the incidences of depression and anxiety were not statistically different among nine lung function trajectories (online Supplementary Table S5). Although not significant, the HRs were over 1 in most of the trajectories, especially those with PRISm at baseline. Compared with consistent normal spirometry, the transition from normal spirometry to PRISm had a higher risk of mortality in participants with depression (HR: 4.27; 95% CI 2.10–8.69) and anxiety (HR: 3.08; 95% CI 1.19–7.94).

## Discussion

In the general population from the UK Biobank study, we found significant associations of PRISm with increased risks of depression, anxiety, and mortality among participants with depression and anxiety. These risks increased non-linearly with the severity of PRISm. The magnitudes of these associations were similar in the phenotypes of lung function impairment and the subtypes of mental disorders. In the lung function trajectory analysis, the transition from normal spirometry to PRISm showed a higher risk of mortality in participants with depression and anxiety.

PRISm is a new lung function impairment phenotype that increases the risks of multiple adverse outcomes. Population-based studies conducted in Europe, the United States and Japan revealed that PRISm was associated with increased all-cause and cause-specific mortality (Higbee et al., 2022; Wan et al., 2018, 2021; Washio et al., 2022; Wijnant et al., 2020). Recent findings also reported the increased risks of developing diabetes, CVD and frailty in individuals with PRISm (He et al., 2023; Krishnan et al., 2023; Li et al., 2023a; Zheng et al., 2023). An interesting phenomenon noted in most of these studies was the similar risks of PRISm and COPD on these adverse outcomes, which was consistent with our findings regarding depression and anxiety. Therefore, PRISm and COPD may have similar comorbidities despite different lung function impairment phenotypes, emphasizing the importance of identifying PRISm.

Previous studies have examined the associations of COPD with depression and anxiety, most of which were cross-sectional designs (Matte et al., 2016). A meta-analysis of six longitudinal studies showed that COPD increased the risk of depression by 69% (Atlantis, Fahey, Cochrane, & Smith, 2013). Another longitudinal study in China observed that chronic lung disease was associated with a 17% increased risk of developing depressive symptoms (Zheng et al., 2022). In addition to confirming and extending prior knowledge about COPD, we comprehensively evaluated the associations of PRISm with depression, anxiety and mortality among participants with depression and anxiety. Our study supported the hypothesis that non-obstructive lung function abnormality could also lead to adverse outcomes of depression and anxiety. More importantly, the potentially reverse *J* shaped associations suggested the accelerated progression of depression and anxiety with the decrease of FEV_1_% predicted in PRISm, highlighting the necessity of preventing PRISm progression. To the best of our knowledge, this is the first study to validate the associations of baseline category and trajectory of PRISm with adverse outcomes of depression and anxiety.

The underlying mechanisms linking PRISm with depression and anxiety have not been understood completely, but several mechanisms may explain the associations. Lung function impairment can cause chronic systemic inflammation and chronic hypoxemia, which may restrict the function of brain cells and induce symptoms of depression and anxiety (Barnes, 2016; Giltay, Nissinen, Giampaoli, Zitman, & Kromhout, 2010). Lung function decline, together with extra-pulmonary comorbidities of PRISm reported in previous studies, can change lifestyle and impair quality of life (e.g. more sedentary, less mobility, dyspnea, pain), resulting in depression and anxiety disorders (Ali et al., 2010; Li, Ge, Greene, & Dunbar-Jacob, 2019; Vreijling et al., 2023). In addition, similar risk factors may also explain the associations of PRISm with depression and anxiety. Further studies are needed to clarify the underlying mechanisms.

Several limitations should be acknowledged in the current study. First, the diagnosis of PRISm and AO with pre-bronchodilator spirometry may overestimate the prevalences of diseases in individuals with reversible airflow obstruction. Second, depression and anxiety in some participants were defined based on self-reported diagnoses, which may introduce recall bias. Third, the limited sample size of some subtypes prevented further exploration on the heterogeneity of associations between lung function impairment phenotype and subtypes of mental disorders. Fourth, the follow-up period was not long enough for the participants with repeated spirometry, possibly leading to an underestimate of the differences between lung function trajectories. Finally, although we adjusted for several important confounders, residual or unmeasured confounding may remain.

In conclusion, this prospective study reveals that PRISm is associated with an increased risk of depression and anxiety, as well as a higher mortality of participants with depression and anxiety. Individuals with PRISm show a similar risk of depression and anxiety as those with AO. Therefore, these findings emphasize the importance of recognizing PRISm and lung function rehabilitation in preventing the adverse outcomes of depression and anxiety.

## Supporting information

Yang et al. supplementary material Yang et al. supplementary material

## Supplementary material

The supplementary material for this article can be found at https://doi.org/10.1017/S0033291724002162
