# Supplementary material for: The association between preserved ratio impaired spirometry and adverse outcomes of depression and anxiety: evidence from the UK Biobank
Source: Psychol Med. 2024 Sep 26;54(12):3530–8. doi: 10.1017/S0033291724002162 (PMC11496235; doi:10.1017/S0033291724002162)
Supplement: Yang et al. supplementary material [file S0033291724002162sup001.docx]

**The association between preserved ratio impaired spirometry and** **adverse outcomes of depression and anxiety: evidence from the UK Biobank**

Kai Yang^1, #^, Lingwei Wang^1, #^, Jun Shen^2, #^, Shuyu Chen^1^, Yuanyuan Liu^1^, Rongchang Chen^1, *^

^1^ Department of Pulmonary and Critical Care Medicine, Shenzhen Institute of Respiratory Diseases, Shenzhen People's Hospital (First Affiliated Hospital of Southern University of Science and Technology, Second Clinical Medical College of Jinan University), Shenzhen, 518001, China

^2^ Department of Orthopedics, the Seventh Affiliated Hospital, Sun Yat-Sen University, Shenzhen, 518000, China

^#^ These authors contributed equally to this work.

^*^Corresponding author: Rongchang Chen; E-mail: chen.rc@szhospital.com.

**Supplementary Method**

**R code for penalized cubic splines fitted in Cox proportional hazard models**

mfit <- coxph(Surv(death_time,status) ~ pspline(fevp, df=4)+age+sex+income+

race+smoke+drink+bmi+physical+diabete_baseline+

hyper_baseline+cvd_baseline, data=data)

ptemp<-termplot(mfit, se=TRUE, plot = F)

fevterm <- ptemp$fevp

center <- with(fevterm, y[x==80])

ytemp <- fevterm$y + outer(fevterm$se, c(0, -1.96, 1.96), '*')

plot(c(0, 200), c(0,2), type = "n",xlab = "",ylab="",axes=F)

polygon(c(-100,-100,80,80),c(-100,3,3,-100),col = "lightblue1")

par(new=T,cex=1.5,mgp=c(2,0.6,0),cex.lab=1.3)

matplot(fevterm$x, exp(ytemp - center),

type='l', lty=c(1,2,2), col="#0072B5FF",lwd = c(3,2,2),

xlab="FEV1 % predicted", ylab="HR for death in depression patients")

abline(h=1,lty=2)

text(37,7.3,labels="PRISm",cex=1.3)

text(145,7.3,labels="Normal spirometry",cex=1.3)

Supplementary Table S1. The definition or classification of covariates in this study.

| Covariate | Definition or classification |
| --- | --- |
| Age, years | Continuous |
| Sex | Male, Female |
| Ethnicity | White, Black, Other |
| BMI, kg/m^3^ | Continuous |
| Smoking status | Never, Previous, Current |
| Drinking status | Never, Previous, Current |
| Income level, £ | <31000, 31000~ |
| Physical activity, days/week | 0, 1~2, 3~7 |
| Hypertension | ICD-10 codes I10-I13 and I15 |
| Diabetes | ICD-10 codes E10-E14 |
| Cardiovascular disease | ICD-10 codes I5-I9, I11, I13, I20-I28 and I30-I52 |

Supplement Table S2. Prevalences and incidences of outcome events in different populations.

| Event | Normal | PRISm | AO |
| --- | --- | --- | --- |
| Total population |  |  |  |
| Prevalence of depression at baseline | 21796(7.9) | 3628(9.3) ^ab^ | 4282(7.9) |
| Depressive episode | 20948(7.6) | 3472(8.9) ^ab^ | 4105(7.6) |
| Recurrent depressive disorder | 1304(0.5) | 237(0.6) ^ab^ | 281(0.5) |
| Persistent mood disorder | 326(0.1) | 65(0.2) ^ab^ | 70(0.1) |
| Other mood disorder | 52(0.0) | 5(0.0) | 7(0.0) |
| Unspecified mood disorder | 310(0.1) | 51(0.1) | 46(0.1) |
| Prevalence of anxiety at baseline | 9907(3.6) | 1530(3.9) ^ab^ | 1960(3.6) |
| Phobic anxiety disorder | 794(0.3) | 134(0.3) | 149(0.3) |
| Other anxiety disorder | 9316(3.4) | 1439(3.7) ^ab^ | 1848(3.4) |
| Population 1 |  |  |  |
| Incidence of depression during follow-up | 10118(4.0) | 1865(5.3) ^ab^ | 2318(4.6) |
| Depressive episode | 9928(3.9) | 1856(5.2) ^ab^ | 2304(4.6) |
| Recurrent depressive disorder | 586(0.2) | 98(0.3) | 131(0.2) |
| Persistent mood disorder | 131(0.1) | 25(0.1) | 28(0.1) |
| Other mood disorder | 45(0.0) | 6(0.0) | 15(0.0) |
| Unspecified mood disorder | 221(0.1) | 40(0.1) ^b^ | 31(0.1) |
| Incidence of anxiety during follow-up | 11770(4.4) | 2055(5.5) ^ab^ | 2630(5.0) |
| Phobic anxiety disorder | 1245(0.5) | 281(0.7) ^ab^ | 284(0.5) |
| Other anxiety disorder | 11025(4.1) | 1891(5.1) ^ab^ | 2455(4.7) |
| Population 2 |  |  |  |
| Incidence of death in participants with depression | 1441(6.6) | 452(12.5) ^ab^ | 662(15.5) |
| Depressive episode | 1389(6.6) | 428(12.3) ^ab^ | 631(15.4) |
| Recurrent depressive disorder | 94(7.2) | 32(13.5) ^ab^ | 57(20.3) |
| Persistent mood disorder | 27(8.3) | 7(10.8) | 12(17.1) |
| Other mood disorder | 4(7.7) | 2(40.0) | 1(14.3) |
| Unspecified mood disorder | 17(5.5) | 6(11.8) | 6(13.0) |
| Incidence of death in participants with anxiety | 590(6.0) | 186(12.2) ^ab^ | 312(15.9) |
| Phobic anxiety disorder | 47(5.9) | 18(13.4) ^a^ | 23(15.4) |
| Other anxiety disorder | 555(6.0) | 172(12.0) ^ab^ | 296(16.0) |

^a^ *P*<0.05 for the comparison of PRISm and normal spirometry.

^b^ *P*<0.05 for the comparison of PRISm and AO.

Supplement Table S3. Demographic information of the subpopulation.

|  | Normal to normal | Normal to PRISm | Normal to AO | PRISm to normal | PRISm to PRISm | PRISm to AO | AO to normal | AO to PRISm | AO to AO |
| --- | --- | --- | --- | --- | --- | --- | --- | --- | --- |
| **N** | 24457 | 1286 | 2678 | 1172 | 1137 | 345 | 1296 | 252 | 2696 |
| **Age** | 54.2±7.5 | 54.5±7.6 | 56.9±7.5 | 53.5±7.3 | 54.1±7.8 ^c^ | 56.7±7.6 | 56.4±7.4 | 55.6±7.7 | 57.3±7.3 |
| **Sex** |  |  |  |  |  |  |  |  |  |
| Male | 11615(47.5) | 565(43.9) ^a^ | 1318(49.2) | 566(48.3) | 531(46.7) | 161(46.7) | 743(57.3) | 130(51.6) | 1715(63.6) |
| Female | 12842(52.5) | 721(56.1) ^a^ | 1360(50.8) | 606(51.7) | 606(53.3) | 184(53.3) | 553(42.7) | 122(48.4) | 981(36.4) |
| **Ethnicity** |  |  |  |  |  |  |  |  |  |
| White | 23860(97.6) | 1228(95.5) ^a^ | 2606(97.3) | 1114(95.1) | 1034(90.9) ^bc^ | 330(95.7) | 1271(98.1) | 234(92.9) | 2648(98.2) |
| Black | 141(0.6) | 16(1.2) ^a^ | 12(0.5) | 9(0.8) | 18(1.6) ^bc^ | 1(0.3) | 5(0.4) | 4(1.6) | 10(0.4) |
| Other | 456(1.9) | 42(3.3) ^a^ | 60(2.2) | 49(4.2) | 85(7.5) ^bc^ | 14(4.1) | 20(1.5) | 14(5.6) | 38(1.4) |
| **BMI** | 26.5±4.1 | 27.6±4.7 ^a^ | 25.9±4.0 | 28.1±5.3 | 28.5±5.4 ^c^ | 27.2±4.8 | 26±3.7 | 26.8±4.3 | 25.7±3.8 |
| **Smoking status** |  |  |  |  |  |  |  |  |  |
| Never | 15286(62.5) | 788(61.3) ^a^ | 1544(57.7) | 723(61.7) | 688(60.5) | 185(53.6) | 756(58.3) | 149(59.1) | 1336(49.6) |
| Previous | 7945(32.5) | 374(29.1) ^a^ | 930(34.7) | 372(31.7) | 355(31.2) | 126(36.5) | 460(35.5) | 76(30.2) | 1041(38.6) |
| Current | 1226(5.0) | 124(9.6) ^a^ | 204(7.6) | 77(6.6) | 94(8.3) | 34(9.9) | 80(6.2) | 27(10.7) | 319(11.8) |
| **Drinking Status** |  |  |  |  |  |  |  |  |  |
| Never | 552(2.3) | 47(3.7) ^a^ | 75(2.8) | 32(2.7) | 54(4.8) ^b^ | 11(3.2) | 29(2.2) | 5(2.0) | 56(2.1) |
| Previous | 471(1.9) | 31(2.4) ^a^ | 70(2.6) | 33(2.8) | 26(2.3) ^b^ | 10(2.9) | 23(1.8) | 4(1.6) | 73(2.7) |
| Current | 23434(95.8) | 1208(93.9) ^a^ | 2533(94.6) | 1107(94.5) | 1057(93.0) ^b^ | 324(93.9) | 1244(96.0) | 243(96.4) | 2567(95.2) |
| **Physical activity** |  |  |  |  |  |  |  |  |  |
| 0 days/week | 2865(11.7) | 195(15.2) ^a^ | 295(11.0) | 178(15.2) | 171(15.0) | 43(12.5) | 137(10.6) | 28(11.1) | 297(11.0) |
| 1–3 days/week | 10487(42.9) | 516(40.1) ^a^ | 1107(41.3) | 523(44.6) | 500(44.0) | 145(42.0) | 528(40.7) | 109(43.3) | 1124(41.7) |
| 3–7 days/week | 11105(45.4) | 575(44.7) ^a^ | 1276(47.7) | 471(40.2) | 466(41.0) | 157(45.5) | 631(48.7) | 115(45.6) | 1275(47.3) |
| **Income level** |  |  |  |  |  |  |  |  |  |
| <31000 | 7693(31.5) | 464(36.1) ^a^ | 1026(38.3) | 379(32.3) | 425(37.4) ^b^ | 139(40.3) | 474(36.6) | 102(40.5) | 1043(38.7) |
| 31000~ | 16764(68.5) | 822(63.9) ^a^ | 1652(61.7) | 793(67.7) | 712(62.6) ^b^ | 206(59.7) | 822(63.4) | 150(59.5) | 1653(61.3) |
| **First FEV_1_/FVC** | 78.6±4.1 | 77.8±4.1 ^a^ | 75.9±4.3 | 77.2±4.3 | 77.0±4.3 ^c^ | 74.0±3.7 | 65.8±6.2 | 66.4±4.7 | 64.5±5.2 |
| **Second FEV_1_/FVC** | 78.0±4.0 | 76.3±4.3 ^a^ | 62.1±9.8 | 77.8±4.0 | 76.6±4.3 ^bc^ | 64.3±8.1 | 74.1±3.7 | 73.5±3.9 | 63.6±6.5 |
| **First FEV_1_ % predicted** | 101.4±12.8 | 89.0±8.6 ^a^ | 99.3±12.3 | 73.5±7.7 | 72.5±6.8 ^b^ | 72.8±7.9 | 91.0±19.8 | 74.3±10.6 | 82.5±16.4 |
| **Second FEV_1_ % predicted** | 101.2±12.0 | 74.4±6.0 ^a^ | 82.8±23.6 | 90±8.8 | 71.9±6.9 ^bc^ | 70±14.9 | 97.2±11.8 | 71.9±8.9 | 78.9±17.9 |
| **Hypertension at baseline** | 4970(20.3) | 342(26.6) ^a^ | 578(21.6) | 284(24.2) | 330(29.0) ^b^ | 93(27.0) | 269(20.8) | 83(32.9) | 617(22.9) |
| **Diabetes at baseline** | 378(1.6) | 32(2.5) ^a^ | 36(1.3) | 29(2.5) | 59(5.2) ^b^ | 12(3.5) | 16(1.2) | 4(1.6) | 54(2.0) |
| **CVD at baseline** | 1288(5.3) | 100(7.8) ^a^ | 167(6.2) | 85(7.3) | 101(8.9) | 30(8.7) | 71(5.5) | 15(6.0) | 206(7.6) |

^a^ P<0.05 compared with participants with persistent normal spirometry.

^b^ P<0.05 compared with participants with PRISm at baseline and normal spirometry at follow-up.

^c^ P<0.05 compared with participants with PRISm at baseline and AO at follow-up.

Supplement Table S4. Prevalences and incidences of outcome events in the subpopulation.

|  | Normal to normal | Normal to PRISm | Normal to AO | PRISm to normal | PRISm to PRISm | PRISm to AO | AO to normal | AO to PRISm | AO to AO | P |
| --- | --- | --- | --- | --- | --- | --- | --- | --- | --- | --- |
| Total population |  |  |  |  |  |  |  |  |  |  |
| Prevalence of depression at baseline | 2988(12.2) | 185(14.4) | 317(11.8) | 191(16.3) | 140(12.3) | 52(15.1) | 137(10.6) | 25(9.9) | 294(10.9) | <0.01 |
| Depressive episode | 2905(11.9) | 180(14) | 312(11.7) | 188(16) | 137(12.1) | 50(14.5) | 134(10.3) | 24(9.5) | 285(10.6) |  |
| Recurrent depressive disorder | 118(0.5) | 11(0.9) | 11(0.4) | 12(1) | 8(0.7) | 2(0.6) | 6(0.5) | 1(0.4) | 15(0.6) |  |
| Persistent mood disorder | 35(0.1) | 1(0.1) | 5(0.2) | 2(0.2) | 4(0.4) | 3(0.9) | 5(0.4) | 2(0.8) | 6(0.2) |  |
| Other mood disorder | 10(0) | 1(0.1) | 2(0.1) | 0(0) | 0(0) | 0(0) | 0(0) | 0(0) | 1(0) |  |
| Unspecified mood disorder | 49(0.2) | 2(0.2) | 2(0.1) | 0(0) | 2(0.2) | 1(0.3) | 2(0.2) | 0(0) | 3(0.1) |  |
| Prevalence of anxiety at baseline | 1690(6.9) | 117(9.1) | 180(6.7) | 92(7.9) | 97(8.5) | 24(7.0) | 93(7.2) | 23(9.1) | 168(6.2) | 0.02 |
| Phobic anxiety disorder | 92(0.4) | 10(0.8) | 9(0.3) | 7(0.6) | 4(0.4) | 4(1.2) | 8(0.6) | 3(1.2) | 11(0.4) |  |
| Other anxiety disorder | 1618(6.6) | 107(8.3) | 175(6.5) | 89(7.6) | 94(8.3) | 24(7) | 86(6.6) | 20(7.9) | 160(5.9) |  |
| Population 1 |  |  |  |  |  |  |  |  |  |  |
| Incidence of depression during follow-up | 185(0.9) | 8(0.7) | 24(1.0) | 13(1.3) | 14(1.4) | 5(1.7) | 8(0.7) | 2(0.9) | 25(1.0) | 0.35 |
| Depressive episode | 190(0.9) | 8(0.7) | 24(1) | 14(1.4) | 14(1.4) | 5(1.7) | 8(0.7) | 2(0.8) | 25(1) |  |
| Recurrent depressive disorder | 4(0) | 1(0.1) | 0(0) | 0(0) | 0(0) | 0(0) | 0(0) | 0(0) | 0(0) |  |
| Persistent mood disorder | 1(0) | 0(0) | 0(0) | 0(0) | 0(0) | 0(0) | 0(0) | 0(0) | 0(0) |  |
| Other mood disorder | 0(0) | 0(0) | 0(0) | 0(0) | 0(0) | 0(0) | 0(0) | 0(0) | 0(0) |  |
| Unspecified mood disorder | 2(0) | 0(0) | 0(0) | 0(0) | 0(0) | 0(0) | 0(0) | 0(0) | 0(0) |  |
| Incidence of anxiety during follow-up | 346(1.5) | 24(2.1) | 35(1.4) | 18(1.7) | 18(1.7) | 8(2.5) | 18(1.5) | 3(1.3) | 44(1.7) | 0.73 |
| Phobic anxiety disorder | 31(0.1) | 1(0.1) | 1(0) | 3(0.3) | 5(0.4) | 0(0) | 1(0.1) | 0(0) | 4(0.2) |  |
| Other anxiety disorder | 327(1.4) | 24(2) | 35(1.4) | 17(1.5) | 15(1.4) | 8(2.4) | 18(1.5) | 3(1.3) | 43(1.7) |  |
| Population 2 |  |  |  |  |  |  |  |  |  |  |
| Incidence of death in participants with depression | 38(1.3) | 10(5.4) | 8(2.5) | 5(2.6) | 5(3.6) | 2(3.9) | 2(1.5) | 1(4.0) | 7(2.4) | <0.01 |
| Depressive episode | 32(1.4) | 6(4.2) | 5(2) | 3(2.1) | 5(4.4) | 1(2.3) | 2(1.9) | 1(6.7) | 5(2.2) |  |
| Recurrent depressive disorder | 0(0) | 1(12.5) | 0(0) | 0(0) | 1(33.3) | 0(0) | 1(20) | 0(0) | 3(0) |  |
| Persistent mood disorder | 0(0) | 0(0) | 0(0) | 0(0) | 0(0) | 1(33.3) | 1(25) | 0(0) | 0(0) |  |
| Other mood disorder | 0(0) | 0(0) | 0(0) | 0(0) | 0(0) | 0(0) | 0(0) | 0(0) | 0(0) |  |
| Unspecified mood disorder | 0(0) | 0(0) | 0(0) | 0(0) | 1(50) | 0(0) | 0(0) | 0(0) | 0(0) |  |
| Incidence of death in participants with anxiety | 23(1.4) | 6(5.1) | 2(1.1) | 3(3.3) | 1(1.0) | 0(0.0) | 4(4.3) | 2(8.7) | 3(1.8) | <0.01 |
| Phobic anxiety disorder | 0(0) | 2(50) | 0(0) | 0(0) | 0(0) | 0(0) | 0(0) | 0(0) | 0(0) |  |
| Other anxiety disorder | 16(1.5) | 3(4.6) | 1(0.8) | 1(1.8) | 1(1.7) | 0(0) | 1(1.8) | 2(14.3) | 1(0.9) |  |

Supplement Table S5. Association of lung function trajectories with depression and anxiety.

| Population | Trajectory | Crude model | |  | Adjusted model^a^ | |
| --- | --- | --- | --- | --- | --- | --- |
|  |  | HR (95% CI) | *P* |  | HR (95% CI) | *P* |
| Population 1 |  |  |  |  |  |  |
| Depression | Normal to normal | Reference |  |  | Reference |  |
|  | Normal to PRISm | 0.91(0.45, 1.85) | 0.80 |  | 0.79(0.39, 1.61) | 0.51 |
|  | Normal to AO | 1.24(0.81, 1.90) | 0.32 |  | 1.28(0.83, 1.96) | 0.26 |
|  | PRISm to normal | 1.45(0.82, 2.54) | 0.20 |  | 1.21(0.68, 2.14) | 0.51 |
|  | PRISm to PRISm | 1.66(0.96, 2.86) | 0.07 |  | 1.42(0.82, 2.45) | 0.21 |
|  | PRISm to AO | 2.01(0.83, 4.88) | 0.12 |  | 1.85(0.76, 4.50) | 0.18 |
|  | AO to normal | 0.81(0.40, 1.64) | 0.55 |  | 0.85(0.42, 1.73) | 0.66 |
|  | AO to PRISm | 1.05(0.26, 4.23) | 0.95 |  | 0.99(0.25, 3.98) | 0.98 |
|  | AO to AO | 1.25(0.82, 1.90) | 0.29 |  | 1.27(0.83, 1.95) | 0.27 |
| Anxiety | Normal to normal | Reference |  |  | Reference |  |
|  | Normal to PRISm | 1.48(0.98, 2.24) | 0.06 |  | 1.33(0.88, 2.01) | 0.18 |
|  | Normal to AO | 0.98(0.69, 1.39) | 0.91 |  | 0.93(0.66, 1.32) | 0.68 |
|  | PRISm to normal | 1.02(0.63, 1.63) | 0.95 |  | 0.95(0.59, 1.54) | 0.84 |
|  | PRISm to PRISm | 1.16(0.72, 1.86) | 0.54 |  | 1.01(0.63, 1.63) | 0.96 |
|  | PRISm to AO | 1.65(0.82, 3.32) | 0.16 |  | 1.42(0.70, 2.87) | 0.33 |
|  | AO to normal | 1.00(0.62, 1.60) | 0.99 |  | 1.03(0.64, 1.65) | 0.92 |
|  | AO to PRISm | 0.91(0.29, 2.83) | 0.87 |  | 0.85(0.27, 2.64) | 0.77 |
|  | AO to AO | 1.19(0.87, 1.63) | 0.28 |  | 1.17(0.85, 1.61) | 0.34 |
| Population 2 |  |  |  |  |  |  |
| Depression | Normal to normal | Reference |  |  | Reference |  |
|  | Normal to PRISm | 4.88(2.43, 9.79) | <0.01 |  | 4.27(2.10, 8.69) | <0.01 |
|  | Normal to AO | 2.19(1.02, 4.70) | 0.04 |  | 1.79(0.83, 3.88) | 0.14 |
|  | PRISm to normal | 1.94(0.76, 4.93) | 0.16 |  | 1.66(0.65, 4.26) | 0.29 |
|  | PRISm to PRISm | 2.97(1.17, 7.54) | 0.02 |  | 2.52(0.97, 6.54) | 0.06 |
|  | PRISm to AO | 2.69(0.65, 11.16) | 0.17 |  | 1.58(0.37, 6.69) | 0.54 |
|  | AO to normal | 1.22(0.29, 5.05) | 0.79 |  | 0.98(0.24, 4.09) | 0.98 |
|  | AO to PRISm | 3.74(0.51, 27.24) | 0.19 |  | 3.60(0.49, 26.80) | 0.21 |
|  | AO to AO | 1.96(0.88, 4.40) | 0.10 |  | 1.16(0.50, 2.68) | 0.72 |
| Anxiety | Normal to normal | Reference |  |  | Reference |  |
|  | Normal to PRISm | 3.96(1.61, 9.74) | <0.01 |  | 3.08(1.19, 7.94) | 0.02 |
|  | Normal to AO | 0.89(0.21, 3.76) | 0.87 |  | 0.65(0.15, 2.79) | 0.56 |
|  | PRISm to normal | 2.48(0.75, 8.27) | 0.14 |  | 2.13(0.60, 7.52) | 0.24 |
|  | PRISm to PRISm | 0.77(0.10, 5.67) | 0.79 |  | 0.52(0.07, 3.93) | 0.53 |
|  | PRISm to AO | - | - |  | - | - |
|  | AO to normal | 3.28(1.13, 9.47) | 0.03 |  | 2.90(0.99, 8.51) | 0.05 |
|  | AO to PRISm | 6.06(1.43, 25.73) | 0.01 |  | 4.01(0.91, 17.64) | 0.07 |
|  | AO to AO | 1.44(0.43, 4.8) | 0.55 |  | 0.93(0.27, 3.16) | 0.90 |

^a^ Adjusted for age, sex, ethnicity, BMI, smoking status, drinking status, income level, physical activity, hypertension, diabetes and CVD.


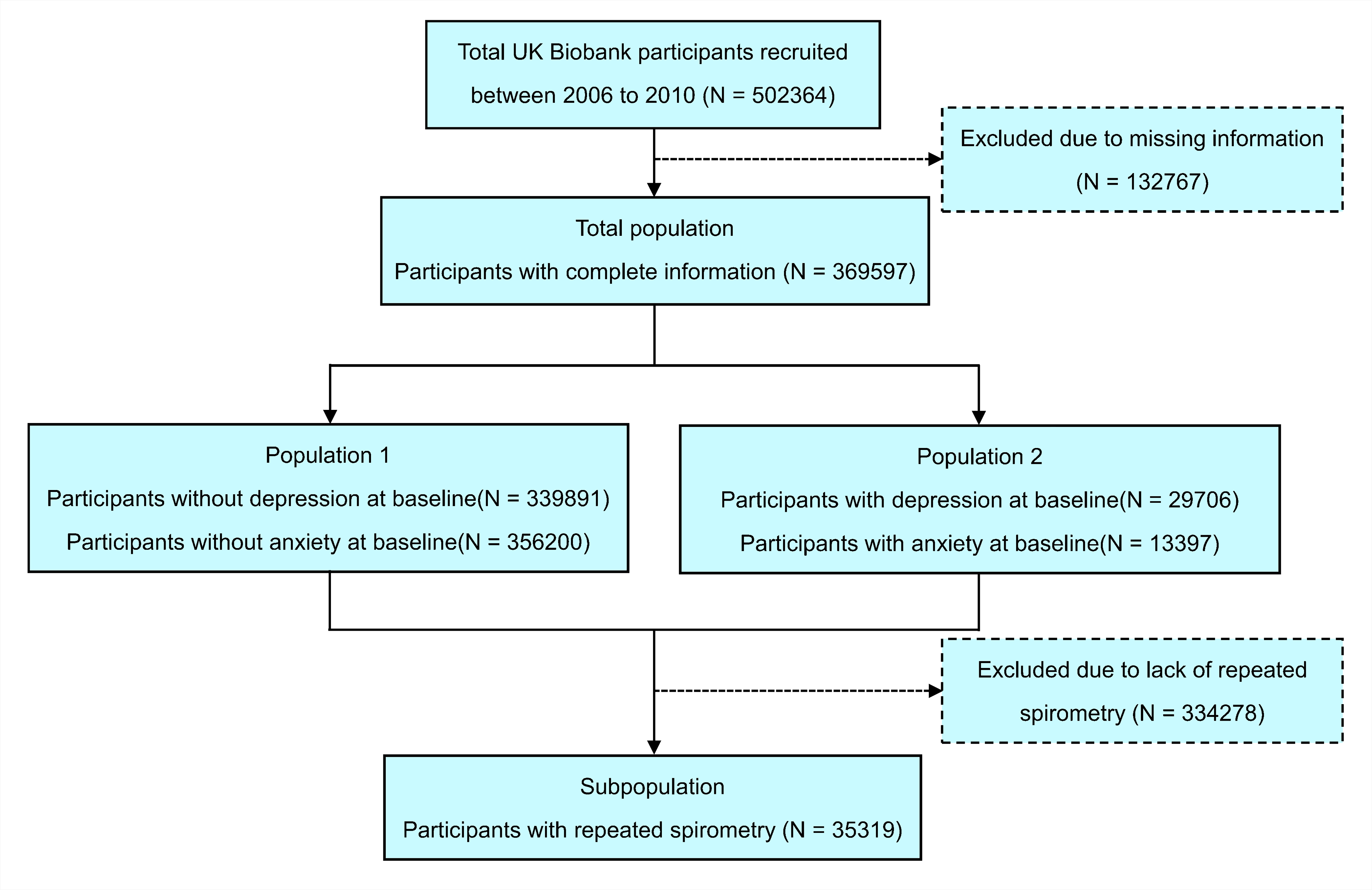


Supplementary Fig. S1. Flowchart of participant selection in this study.


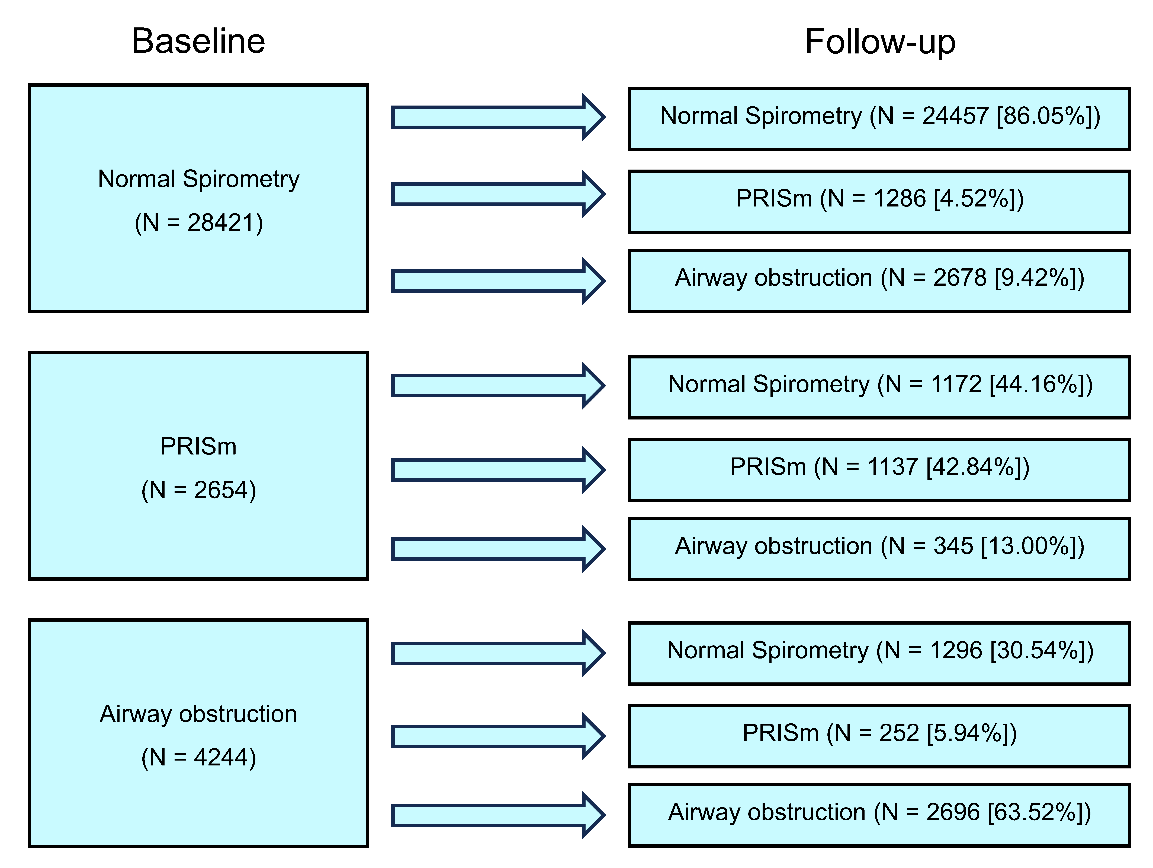


Supplementary Fig. S2. Lung function trajectories from baseline to follow-up.
